# Combining glucagon-like peptide-1 receptor agonists (GLP-1RAs) and sodium-glucose cotransporter-2 inhibitors (SGLT2is) in patients with type 2 diabetes mellitus (T2DM)

**DOI:** 10.1186/s12933-023-01798-4

**Published:** 2023-04-01

**Authors:** Pierre Gourdy, Patrice Darmon, François Dievart, Jean-Michel Halimi, Bruno Guerci

**Affiliations:** 1 Endocrinology, Diabetology and Nutrition Department, Toulouse University Hospital, Toulouse, France; 2 Institute of Metabolic and Cardiovascular Diseases, UMR1297 INSERM/UPS, Toulouse University, Toulouse, France; 3 Aix Marseille University, INSERM, INRA, C2VN, Marseille, France; 4 Department of Cardiology, Villette Private Hospital, Dunkirk, France; 5 Department of Nephrology, Tours University Hospital, Tours, France; 6 EA4245, Tours University, Tours, France; 7 Department of Endocrinology, Diabetology, and Nutrition, Brabois Adult Hospital, University of Lorraine, Vandoeuvre-Lès-Nancy, France

**Keywords:** Glucagon-like peptide-1 receptor agonists, Sodium-glucose cotransporter-2 inhibitors, Type 2 diabetes mellitus, Combination therapy, Cardiovascular protection

## Abstract

Due to their cardiovascular protective effect, glucagon-like peptide-1 receptor agonists (GLP-1RAs) and sodium-glucose cotransporter-2 inhibitors (SGLT2is) represent breakthrough therapies for type 2 diabetes mellitus (T2DM). In this review article, we discuss the mechanistic and clinical synergies that make the combined use of GLP-1RAs and SGLT2is appealing in patients with T2DM. Overall, the presented cumulative evidence supports the benefits of GLP-1RA plus SGLT2i combination therapy on metabolic-cardiovascular-renal disease in patients with T2DM, with a low hypoglycemia risk. Accordingly, we encourage the adoption of GLP-1RA plus SGLT2i combination therapy in patients with T2DM and established atherosclerotic cardiovascular disease (ASCVD) or multiple risk factors for ASCVD (i.e., age ≥ 55 years, overweight/obesity, dyslipidemia, hypertension, current tobacco use, left ventricular hypertrophy, and/or proteinuria). Regarding renal effects, the evidence of SGLT2is in preventing kidney failure is more abundant than for GLP-1RAs, which showed a beneficial effect on albuminuria but not on hard kidney endpoints. Hence, in case of persistent albuminuria and/or uncontrolled metabolic risks (i.e., inadequate glycemic control, hypertension, overweight/obesity) on SGLT2i therapy, GLP-1RAs should be considered as the preferential add-on therapy in T2DM patients with chronic kidney disease. Despite the potential clinical benefits of GLP-1RA plus SGLT2i combination therapy in patients with T2DM, several factors may delay this combination to become a common practice soon, such as reimbursement and costs associated with polypharmacy. Altogether, when administering GLP-1RA plus SGLT2i combination therapy, it is important to adopt an individualized approach to therapy taking into account individual preferences, costs and coverage, toxicity profile, consideration of kidney function and glucose-lowering efficacy, desire for weight loss, and comorbidities.

## Introduction

Management of type 2 diabetes mellitus (T2DM) has evolved from a glucocentric to a cardiometabolic approach [1]. Consequently, choosing anti-hyperglycemic therapies with proven cardiovascular and renal benefits is now a cornerstone of T2DM management [2]. Both glucagon-like peptide-1 receptor agonists (GLP-1RAs) and sodium-glucose cotransporter-2 inhibitors (SGLT2is) have individually been shown to reduce cardiovascular and kidney outcomes in patients with T2DM, with a low hypoglycemia risk [3, 4]. Cardiovascular risk reduction seems however to be more pronounced in individuals with T2DM and established atherosclerotic cardiovascular disease (ASCVD) as compared to those without established ASCVD [5].

Overall, both classes are indifferently recommended by the American Diabetes Association (ADA) and the European Association for the Study of Diabetes (EASD) as first-line therapy in patients with T2DM and established ASCVD or multiple ASCVD risk factors to reduce the risk of major adverse cardiovascular events (MACE), such as myocardial infarction, stroke, and cardiovascular death [6, 7]. In such patients, the ADA-EASD 2022 consensus report also recommends the combination of GLP-1RAs and SGLT2is when hemoglobin A1c (HbA1c) target is not reached with a drug from one of the two classes [6].

The ADA and the EASD further recommend SGLT2is as first-line therapy in patients with T2DM and heart failure (HF) to reduce the risk of worsening HF [6, 7]. Moreover, SGLT2is are recommended as first-line therapy in patients with T2DM and chronic kidney disease (CKD). In case of CKD, the use of a GLP-1RA with proven cardiovascular benefit is only recommended in those who have not achieved individualized glycemic targets despite SGLT2i treatment, or in whom SGLT2is are contraindicated or not tolerated [6, 8].

GLP-1RAs and SGLT2is act through distinct and complementary mechanisms of action to exert glycemic control and cardiovascular benefits [4, 9]. In this review, we discuss the mechanistic and clinical synergies that make the combined use of GLP-1RAs and SGLT2is appealing in patients with T2DM.

Our review was based on a systematic PubMed search of randomized controlled trials (RCTs), observational studies, systematic reviews/meta-analyses, literature reviews, and case reports, published up to 2022, evaluating the combined treatment with a GLP-1RA and a SGLT2i in T2DM or prediabetes. The used search terms were: "GLP-1 receptor agonist and SGLT2 inhibitor" OR "SGLT2 inhibitor and GLP-1 receptor agonist" OR "SGLT-2 inhibitor and GLP-1 receptor agonist" OR "GLP-1 receptor agonist and SGLT-2 inhibitor" OR "GLP-1 receptor agonists as add-on to SGLT2 inhibitors" OR "GLP1 receptor agonist and SGLT-2 inhibitor" OR "GLP-1 agonist plus SGLT2 inhibitor" OR "glucagon-like peptide-1 receptor agonist and sodium-glucose cotransporter 2 inhibitor" OR "sodium-glucose cotransporter 2 inhibitor and glucagon-like peptide-1 receptor agonist" OR "SGLT2 inhibitors and GLP1-RA" OR "addition of SGLT2i to GLP-1RA" OR "SGLT-2 inhibitor therapy added to GLP-1 agonist therapy" OR "GLP-1 analogues and SGLT-2 inhibitors".

### Rationale for combining GLP-1RAs and SGLT2is

#### Mechanistic perspective

**Fig. 1:**
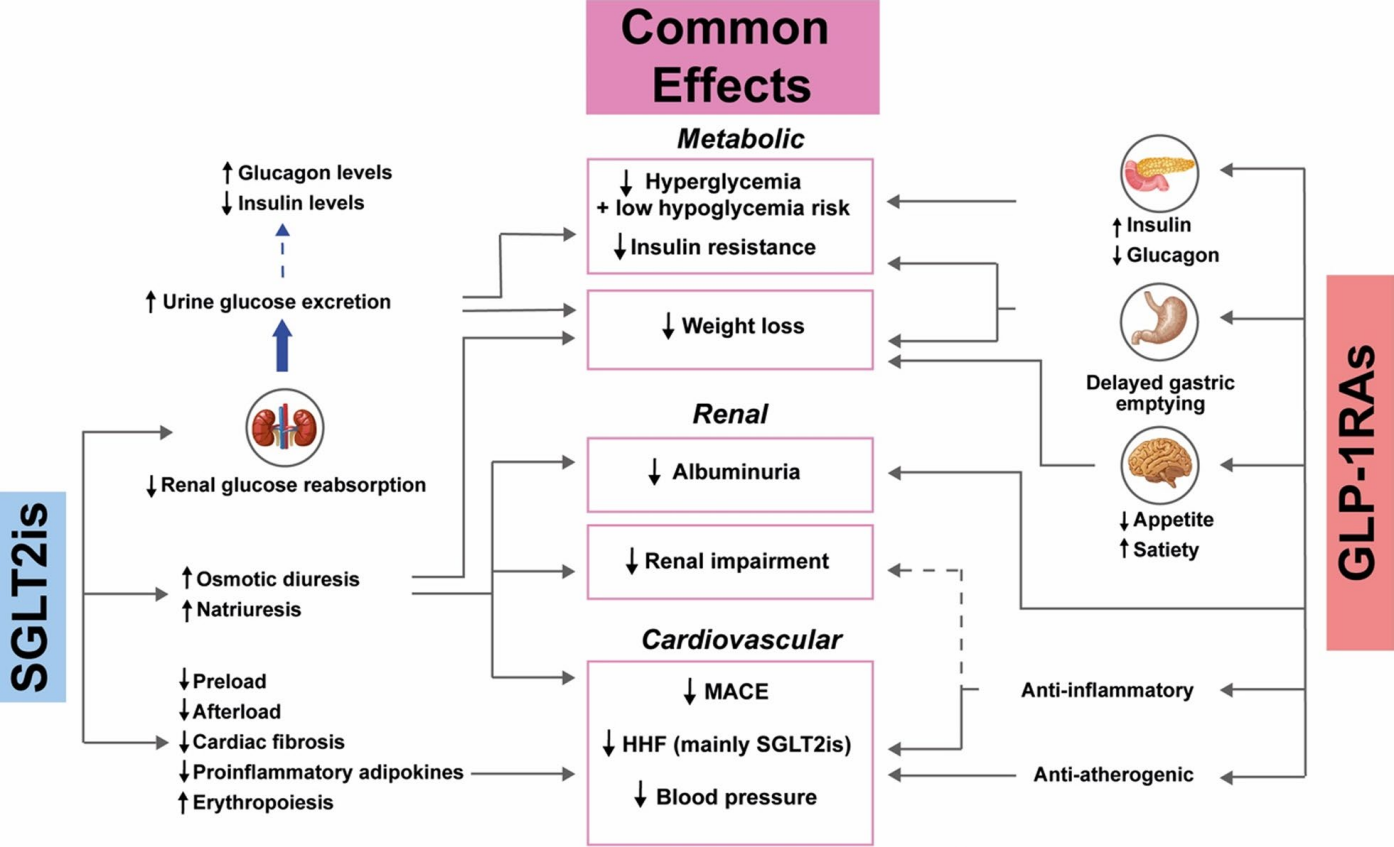
Complementary mechanisms of action of sodium-glucose cotransporter-2 inhibitors (SGLT2is) and glucagon-like peptide-1 receptor agonists (GLP-1RAs). HHF, hospitalization for heart failure; MACE, major adverse cardiovascular events. SGLT2is improve insulin secretion without increasing insulin levels. The black dotted line indicates that there is insufficient clinical evidence to support the beneficial effects of GLP-1RAs on renal impairment [87]

GLP-1RAs and SGLT2is affect glucose metabolism differently (Fig. 1). GLP-1RAs enhance insulin secretion and inhibit glucagon release by the pancreas, resulting in glucose-dependent reductions in plasma glucose. More specifically, GLP-1RAs control postprandial glucose levels through inhibition of hepatic glucose production and delayed gastric emptying [9]. SGLT2is decrease plasma glucose levels through inhibition of renal glucose reabsorption in the proximal tubule, which results in increased glucose excretion by the kidneys. These reductions in plasma glucose lead to improvements in insulin sensitivity and β-cell function [9]. Since GLP-1RAs and SGLT2is act through a glucose-dependent mechanism, they have a low hypoglycemia risk [10].

GLP-1RAs and SGLT2is also provide sustained weight loss via different mechanisms. In addition to delaying gastric emptying, GLP-1RAs exert direct effects on the central nervous system to suppress appetite and promote weight loss [9, 11]. SGLT2is cause weight loss by decreasing body water due to osmotic diuresis and by increasing the excretion of calories in the urine [11].

The mechanisms by which GLP-1RAs and SGLT2is exert their cardiovascular and renal benefits (Fig. 1) also appear to be complementary and mostly independent of their glucose-lowering properties [3, 12–14]. On one hand, the cardiovascular protection afforded by SGLT2is seems to result from their beneficial hemodynamic effects, including improvement in ventricular preload (secondary to natriuresis and osmotic diuresis) and afterload (through blood pressure reduction), but also from improvement of cardiac metabolism (through the switch from utilization of glucose to ketones), inhibition of the myocardial Na^+^/H^+^ exchanger, reduction of cardiac fibrosis and necrosis, reduction of proinflammatory adipokines derived from epicardial and perivascular fat, and stimulation of erythropoiesis (which can facilitate the release of oxygen to ischemic tissues) [1, 4, 14–16]. GLP-1RAs provide different mechanistic effects including anti-oxidative, anti-inflammatory, and anti-atherosclerotic properties [4, 9, 11, 14, 17]. Hence, because some of the cardiovascular benefits of SGLT2is are related to their hemodynamic effects, while those of GLP-1RAs are mainly related to their anti-atherogenic/anti-inflammatory actions, the two classes of medications may produce an additive cardiovascular benefit [11]. More recently, however, an animal study based on a tandem stenosis mouse model showed the ability of SGLT2is to stabilize atherosclerotic plaques, with increased fibrosis, augmented collagen accumulation, and significant upregulation of the expression of vasculoprotective NADPH oxidase 4 [18]. Further research may enhance our understanding of the mechanisms by which the combination of GLP-1RAs and SGLT2is acts to derive a cardiovascular benefit.

#### Clinical perspective

SGLT2is and GLP-1RAs exhibit clinical benefits on glycemic control, systolic blood pressure (SBP), body weight, and dyslipidemia, which may all partly contribute to the cardiovascular protection provided by these two drug classes [4, 19]. Compared to GLP-1RAs, SGLT2is are associated with an overall modest mean weight loss of 2–3 kg [20]. A network meta-analysis [21], conducted in 27,018 patients with obesity or overweight and with or without diabetes mellitus, compared the mean weight loss observed with different GLP-1RAs and demonstrated the following trends, from highest to lowest responses: subcutaneous semaglutide at 2.4 mg weekly: − 9.9 kg, liraglutide at > 1.8 mg daily: − 4.5 kg, subcutaneous semaglutide at < 2.4 mg weekly: − 4.3 kg, oral semaglutide: − 2.7 kg, liraglutide at ≤ 1.8 mg daily: − 2.7 kg, extended-release exenatide: − 2.2 kg, immediate-release exenatide: − 1.8 kg, dulaglutide ≥ 1.5 mg: − 1.4 kg, and lixisenatide: − 0.6 kg [21]. GLP-1RAs also provide overall better glycemic control than SGLT2is, with HbA1c lowering of up to 1.4% for GLP-1RAs and up to 0.9% for SGLT2is [4]. It should be noted that the HbA1c-lowering effect of SGLT2is is diminished in the presence of an impaired renal function, i.e., minor at an estimated glomerular filtration rate (eGFR) of 30–45 mL/min/1.73 m^2^ and absent at an eGFR < 30 mL/min/1.73 m^2^ [22].

To date, no head-to-head RCTs have been performed to compare the effects of GLP-1RAs and SGLT2is on cardiovascular outcomes [22]. Nevertheless, meta-analyses of large-scale cardiovascular outcomes trials (CVOTs) of individual agents have shown that the magnitude of the benefits of GLP-1RAs and SGLT2is on MACE are similar (Table 1). In patients with T2DM, both drug classes reduced the composite of myocardial infarction, stroke, and cardiovascular death (MACE) by 10% to 14%. However, for both drug classes, this treatment effect was mainly restricted to patients with established ASCVD, as no statistically significant effect on MACE was seen in patients without established ASCVD. Data from these meta-analyses are however inconsistent with recent real-world evidence showing that SGLT2is and SGLT2is in combination with GLP-1RAs may be beneficial in the primary prevention of MACE [23].

**Table 1:** Cardiovascular and kidney outcomes reported in selected meta-analyses of CVOTs of GLP-1RAs and SGLT2is

Outcome	GLP-1RAs		SGLT2is	
	HR (95% CI)	p-value	HR (95% CI)	p-value
Meta-analysis by Zelniker et al. [3] of 5 GLP-1RA trials and 3 SGLT2i trials				
MACE	0.88 (0.84–0.94)	< 0.001	0.89 (0.83–0.96)	0.001
MACE in established ASCVD	0.87 (0.82–0.92)	NA	0.86 (0.80–0.93)	NA
MACE without established ASCVD	1.03 (0.87–1.23)	NA	1.00 (0.87–1.16)	NA
HHF	0.93 (0.83–1.04)	0.20	0.69 (0.61–0.79)	< 0.001
Risk of stroke	0.86 (0.77–0.97)	0.012	0.97 (0.86–1.10)	0.64
Kidney outcomes	0.82 (0.75–0.89)	< 0.001	0.62 (0.58–0.67)	< 0.001
Kidney outcomes without macroalbuminuria	0.92 (0.80–1.06)	0.24	0.55 (0.48–0.64)	< 0.001

Outcome	HR (95% CI)	p-value
Meta-analysis by Sattar et al. [29] of 8 GLP-1RA trials		
MACE	0.86 (0.80–0.93)	< 0.0001
MACE in established ASCVD	0.85 (0.78–0.92)	NA
MACE without established ASCVD	0.94 (0.83–1.06)	NA
HHF	0.89 (0.82–0.98)	0.013
Risk of stroke	0.83 (0.76–0.92)	0.0002
Kidney outcomes	0.79 (0.73–0.87)	< 0.0001
Kidney outcomes without macroalbuminuria	0.86 (0.72–1.02)	0.089
Meta-analysis by Lee et al. [85] of 9 GLP-1RA trials		
MACE	0.87 (0.81–0.94)	0.00065
Meta-analysis by McGuire et al. [86] of 6 SGLT2i trials		
MACE	0.90 (0.85–0.95)	NA
MACE in established ASCVD	0.89 (0.84–0.95)	NA
MACE without established ASCVD	0.94 (0.83–1.07)	NA
HHF	0.68 (0.61–0.76)	NA
Kidney outcomes	0.62 (0.56–0.70)	NA

*ASCVD* atherosclerotic cardiovascular disease, *CI* confidence interval, *CVOT* cardiovascular outcome trial, *GLP-1RA* glucagon-like peptide-1 receptor agonist, *HHF* hospitalization for heart failure, *HR* hazard ratio, *MACE* major adverse cardiovascular events, *NA* not available, *SGLT2i* sodium-glucose cotransporter-2 inhibitor

Meta-analyses of CVOTs of GLP-1RAs and SGLT2is have further found that GLP-1RAs reduce the risk of stroke by up to 17%, whereas SGLT2is have no appreciable effect (Table 1). However, a meta-analysis of 8 real-world studies found no significant differences in the risks of MACE (hazard ratio [HR], 0.96; 95% confidence interval [CI], 0.84–1.08), myocardial infarction (HR, 0.95; 95% CI, 0.83–1.10), and stroke (HR, 1.01; 95% CI, 0.93–1.10) among patients with T2DM treated with GLP-1RAs versus SGLT2is [24]. Interestingly, sotagliflozin, a dual SGLT2/SGLT1 inhibitor, significantly reduced the total risk of fatal or non-fatal stroke by 34% (HR, 0.66; 95% CI, 0.48–0.91) in the placebo-controlled, phase III SCORED trial performed in patients with T2DM and CKD [25]. In the same trial, sotagliflozin also reduced the risk of fatal or non-fatal myocardial infarction by 32% (HR, 0.68; 95% CI, 0.52–0.89) compared to placebo [25]. This reduction in the risk of atherosclerotic outcomes might be a unique property of dual SGLT2/SGLT1 inhibitors, since intestinal inhibition of SGLT1 may result in a higher concentration of endogenous GLP-1, thus leading to potentially enhanced cardioprotective properties of dual SGLT2/SGLT1 inhibitors compared with pure SGLT2is [26].

In terms of renal outcomes among patients with T2DM, meta-analyses of CVOTs of GLP-1RAs and SGLT2is have shown that both GLP-1RAs and SGLT2is significantly reduced the composite outcome of macroalbuminuria, worsening of eGFR or serum creatinine, end-stage renal disease, or renal death by up to 21% and 38%, respectively. However, when excluding macroalbuminuria, the effect of GLP-1RAs on kidney outcomes was no longer significant (Table 1). Most recently, in the EMPA-KIDNEY trial [27] performed in 6609 patients with CKD, including 3,569 (54.0%) patients without diabetes, with an eGFR ≥ 20 mL/min/1.73 m^2^, therapy with the SGLT2i empagliflozin led to a 28% lower risk of CKD progression or cardiovascular death than placebo (HR, 0.72; 95% CI, 0.64–0.82; p < 0.001). The benefits of empagliflozin were consistent among patients with or without diabetes and regardless of eGFR [27].

The most notable difference between the two drug classes relates to the risk reduction of hospitalization for heart failure (HHF). In a meta-analysis of 5 RCTs including 21,947 participants, SGLT2is significantly reduced the risk of HHF (HR, 0.72; 95% CI, 0.67–0.78; p < 0.0001) in patients with HF across the full spectrum of ejection fraction, including both outpatients and hospitalized patients [28]. On the other hand, a more modest benefit of GLP-1RAs on HHF has been reported, as highlighted by a meta-analysis of 8 CVOTs of 60,080 patients with T2DM and increased cardiovascular risk or with established cardiovascular disease, in which GLP-1RAs reduced HHF by 11% compared to placebo (HR, 0.89; 95% CI, 0.82–0.98; p = 0.013) [29]. Likewise, another meta-analysis, performed on the same GLP-1RA CVOTs and 60,080 patients, reported a reduction of HHF by 10% with GLP-1RAs compared to placebo (HR, 0.90; 95% CI, 0.83–0.98; p = 0.023) [5].

Overall, GLP-1RAs and SGLT2is reduce the risk of MACE to a similar degree, particularly in patients with established ASCVD. Moreover, SGLT2is have a more marked effect on preventing HHF and progression of CKD, whereas GLP-1RAs may reduce the risk of stroke to a greater extent [3]. Combined treatment with both GLP-1RAs and SGLT2is has thus the potential to yield substantial clinical benefits across a wide range of cardiovascular outcomes among patients with T2DM.

### Clinical trial data of GLP-1RA plus SGLT2i combination therapy

The efficacy and safety of combination therapy with a GLP-1RA and a SGLT2i in patients with T2DM have been investigated in RCTs [10, 30–37], as well as in non-randomized trials [38–41], real-world observational studies [1, 12, 23, 42–50], and post-hoc analyses of CVOTs [13, 51–54]. The combination of GLP-1RAs and SGLT2is has also been clinically evaluated in non-diabetic populations such as adults with obesity [55, 56]. However, so far, there have been no published RCTs specifically designed to evaluate the cardiovascular and renal effects of combining GLP-1RAs with SGLT2is [4]. There is an ongoing large RCT, known as PRECIDENTD (ClinicalTrials.gov ID: https://clinicaltrials.gov/ct2/show/NCT05390892), which will assess the cardiorenal effects of GLP-1RA plus SGLT2i combination therapy compared to both GLP-1RA and SGLT2i monotherapy among 9,000 patients with T2DM and established ASCVD or at high ASCVD risk.

Despite the absence of RCTs dedicated to the study of cardiovascular and renal effects of GLP-1RA plus SGLT2i combination therapy, clinical evidence provides support for combining these two drug classes, as this has the potential to significantly reduce cardiovascular events including HHF and slow CKD progression, without the inconvenience and dangers of hypoglycemia [47].

#### RCTs and meta-analyses

A meta-analysis [57], that included a total of 1,913 adults with T2DM, identified only 7 RCTs which compared the combination of a GLP-1RA and a SGLT2i to placebo or an active control, with 6 out of the 7 trials only reporting short-term (up to 30 weeks) outcomes mainly limited to surrogate measures such as HbA1c, body weight, and blood pressure [57]. The only exception was DURATION-8, a 104-week, active-controlled, phase III trial, evaluating the combination of once-weekly subcutaneous exenatide plus once-daily oral dapagliflozin, simultaneously added to stable metformin monotherapy, in 695 patients with T2DM and poor glycemic control [36]. Overall, the meta-analysis [57] showed that compared with GLP-1RAs alone and SGLT2is alone, GLP-1RA plus SGLT2i combination therapy was associated with a greater reduction in HbA1c, body weight, and SBP (Table 2). Regarding cardiovascular outcomes, although odds ratios (ORs) for death, myocardial infarction, and stroke were not significant in the comparisons of GLP-1RA plus SGLT2i combination with either GLP-1RAs or SGLT2is, these events were too few to allow meaningful conclusions. There was also no trial that reported HHF data. Importantly, the meta-analysis showed that combined therapy did not increase the incidence of severe hypoglycemia compared with either GLP-1RAs (OR, 1.38; 95% CI, 0.14−13.14; 3 trials) or SGLT2is alone (OR, 2.39; 95% CI, 0.47−12.27; 5 trials) [57].

**Table 2:** Metabolic outcomes reported in selected meta-analyses of GLP-1RA plus SGLT2i combination therapy

Outcome	Compared to GLP-1RAs alone		Compared to SGLT2is alone	
	WMD (95% CI)	Nb of RCTs	WMD (95% CI)	Nb of RCTs
Meta-analysis by Mantsiou et al. [57] of 7 RCTs including 1913 adults with T2DM				
HbA1c reduction	− 0.61% (− 1.09% to − 0.14%)	4	− 0.85% (− 1.19% to − 0.52%)	6
Body weight loss	− 2.59 kg (− 3.68 to − 1.51 kg)	3	− 1.46 kg (− 2.94 to 0.03 kg)	5
SBP reduction	− 4.13 mmHg (− 7.28 to − 0.99)	4	− 2.66 mmHg (− 5.26 to − 0.06)	6

Outcome	SMD (95% CI) compared to active control/placebo	p-value
Meta-analysis by Guo et al. (2020) [58] of 5 RCTs and 6 non-RCTs including 1,604 adults with T2DM or obesity		
HbA1c reduction	− 1.32% (− 1.43% to − 1.20%)	< 0.001
Body weight loss	− 0.93 kg (− 1.04 to − 0.83 kg)	< 0.001
SBP reduction	− 1.05 mmHg (− 1.17 to − 0.93 mmHg)	< 0.001

Outcome	SMD (95% CI) compared to GLP-1RAs alone and SGLT2is alone	p-value
Meta-analysis by Li et al. [59] of 8 RCTs including 1,895 adults with T2DM		
HbA1c reduction	− 0.77% (− 1.03% to − 0.50%)	< 0.001
Body weight loss	− 0.36 kg (− 0.50 to − 0.21 kg)	< 0.001
SBP reduction	− 0.33 mmHg (− 0.49 to − 0.17 mmHg)	< 0.001

*CI* confidence interval, *GLP-1RA* glucagon-like peptide-1 receptor agonist, *HbA1c* hemoglobin A1c, *HR* hazard ratio, *Nb* number, *RCT* randomized controlled trial, *SBP* systolic blood pressure, *SGLT2i* sodium-glucose cotransporter-2 inhibitor, *SMD* standardized mean difference, *T2DM* type 2 diabetes mellitus, *WMD* weighted mean difference

Another meta-analysis [58], which included a total of 1,604 participants with T2DM or obesity, found that combination therapy with SGLT2is and GLP-1RAs significantly decreased the incidence of cardiovascular events (reported in 2 RCTs) compared with active control/placebo (relative risk [RR], 0.19; 95% CI, 0.04−0.96). The incidence of severe hypoglycemia (reported in 2 RCTs) was not statistically significant between the combination therapy group and the control group (RR, 1.91; 95% CI, 0.89−4.10) [58].

Most recently, Li et al. [59] pooled data from 8 RCTs performed in 1,895 patients with T2DM, and found that compared with SGLT2i monotherapy and GLP-1RA monotherapy, GLP-1RA and SGLT2i combination therapy reduced HbA1c by 0.77%, with the greatest reduction of 1.75% achieved when semaglutide was added to SGLT2i monotherapy for 30 weeks. Compared to monotherapy, the combination regimen was also associated with a greater decrease in fasting plasma glucose, 2-h postprandial glucose, body weight, low-density lipoprotein cholesterol, and SBP [59] (Table 2).

On the other hand, a 16-week single-center RCT by Ali et al. [35] evaluating the combination of liraglutide and canagliflozin versus each therapy alone in 45 patients with poorly controlled T2DM on metformin showed that although liraglutide and canagliflozin produced an additive effect to reduce body weight and SBP, the incremental reduction in mean HbA1c did not yield an additive effect. The opposing actions of GLP-1RAs and SGLT2is on endogenous glucose production, which is increased by SGLT2is and decreased by GLP-1RAs, may partially explain the observed lack of additive benefit of these two classes on glucose control in patients with T2DM [35].

#### Post-hoc analyses of CVOTs

Among CVOTs evaluating individual agents from the two drug classes, the combined use of GLP-1RAs and SGLT2is was rather limited. In GLP-1RA CVOTs, the prevalence of baseline SGLT2i use ranged from 0% to 15.2%, whereas in SGLT2i CVOTs, the prevalence of baseline GLP-1RA use ranged from 2.5% to 4.4% [12, 13].

A post-hoc analysis of the EXSCEL trial [60], which enrolled a total of 14,752 patients with T2DM and previous cardiovascular disease, propensity-matched 575 participants assigned to once-weekly exenatide plus a SGLT2i to: (1) participants in the placebo arm not taking SGLT2is (n = 572), and to (2) participants in the once-weekly exenatide arm not taking SGLT2i (n = 575) [52]. The risk for MACE with the exenatide plus SGLT2i combination was numerically lower compared with both placebo (adjusted HR, 0.68; 95% CI, 0.39–1.17) and exenatide alone (adjusted HR, 0.85; 95% CI, 0.48–1.49). This reduction was driven by a significant decrease in the risk of cardiovascular death compared with placebo (adjusted HR, 0.17; 95% CI, 0.04–0.77) and exenatide alone (adjusted HR, 0.21; 95% CI, 0.05–0.93), as the incidences of non-fatal MI and non-fatal stroke were similar across all comparison groups. All-cause mortality was also significantly reduced with exenatide plus SGLT2i combination therapy compared with placebo (adjusted HR, 0.38; 95% CI, 0.16–0.90) and exenatide alone (adjusted HR, 0.41; 95% CI, 0.17–0.95), with no increase in the risk of serious hypoglycemia versus placebo (adjusted HR, 0.67; 95% CI, 0.26–1.76) or exenatide alone (adjusted HR, 0.77; 95% CI, 0.30–1.99). Exenatide plus SGLT2i combination therapy also significantly improved estimated eGFR slope compared with placebo (+ 1.94 mL/min/1.73 m^2^/year; 95% CI, 0.94–2.94; p < 0.001) and exenatide alone (+ 2.38 mL/min/1.73 m^2^/year; 95% CI, 1.40–3.35; p < 0.001) [52].

The results of the EXCEL post-hoc analysis [52] are complementary to the post-hoc analyses [54] from the DECLARE-TIMI 58 CVOT [61], which assessed the cardiorenal outcomes of dapagliflozin versus placebo in a total of 17,160 patients with T2DM and established ASCVD or multiple cardiovascular risk factors, of whom 750 (4.4%) used GLP-1RAs at baseline [54]. While the benefits of dapagliflozin on MACE were generally consistent regardless of baseline GLP-1RA use, the combination of dapagliflozin and GLP-1RAs resulted in a significantly greater reduction in HHF relative to placebo (HR, 0.20; 95% CI, 0.07–0.60) compared with those not being treated with a GLP-1RA at baseline (HR, 0.77; 95% CI, 0.64–0.92; p for interaction = 0.014). As for renal outcomes (≥ 40% decrease in eGFR, end-stage renal disease, or renal death), DECLARE-TIMI 58 also showed that the benefit of dapagliflozin compared to placebo was consistent in baseline GLP-1RA users (HR, 0.36; 95% CI, 0.11–1.15) versus non-users (HR, 0.54; 95% CI, 0.43–0.67; p for interaction = 0.49) [54].

Another post-hoc analysis of interest is that of the AMPLITUDE-O CVOT [62], which also suggested that the beneficial effects of GLP-1RAs were independent of those provided by concurrent SGLT2i therapy [13]. AMPLITUDE-O evaluated the efficacy and safety of the exendin-based GLP-1RA, efpeglenatide, compared to placebo, in a total of 4,076 patients with T2DM at a high risk for cardiovascular events, of whom 618 (15.2%) used a SGLT2i at baseline [13, 62]. Over a median follow-up of 1.8 years, the effect (HR [95% CI]) of efpeglenatide versus placebo in the absence and presence of baseline SGLT2is, respectively, on MACE (0.74 [0.58–0.94] and 0.70 [0.37–1.30]), renal outcomes (0.70 [0.59–0.83] and 0.52 [0.33–0.83]), and HHF (0.70 [0.42–1.17] and 0.23 [0.05–0.97]) did not statistically differ by baseline SGLT2i use (p values for all interactions ≥ 0.35). Of note, when examining the effects of efpeglenatide on the incidence of MACE in patients taking and not taking SGLT2is at baseline, there were 4.0 and 5.4 MACE per 100 patient-years in the efpeglenatide and placebo groups, respectively, among those not using baseline SGLT2is. This incidence however dropped to 3.4 and 4.7 MACE per 100 patient-years in the efpeglenatide and placebo groups, respectively, among baseline SGLT2i users, supporting combined GLP-1RA and SGLT2i therapy [13]. Furthermore, baseline SGLT2i use did not modify the effects of efpeglenatide on blood pressure, heart rate, body weight, low-density lipoprotein cholesterol, eGFR, and urinary albumin-to-creatinine ratio over time (p values for all interactions ≥ 0.08). The frequency of adverse events (AEs) was also not influenced by baseline SGLT2i use [13].

Overall, these post-hoc analyses of different CVOTs support the concept that the combination of GLP-1RAs plus SGLT2is may be well-tolerated and may provide additional benefits to GLP-1RAs or SGLT2is alone in terms of cardiovascular and renal protection and mortality, without an increase in the risk of hypoglycemia [13, 52, 54].

#### Observational studies

Accumulating real-world evidence supports the combined use of GLP-1RAs and SGLT2is to reduce the risk of MACE. Using United States insurance claims databases between April 2013 and June 2018, Dave et al. identified patients with T2DM who were already taking GLP-1RAs and who had added either SGLT2is or sulfonylurea [12]. After 1:1 propensity score matching on > 95 variables, 12,584 patients in each group were analyzed for the primary outcomes of a composite cardiovascular endpoint (comprising MI, stroke, and all-cause mortality) and HHF. Compared with the initiation of sulfonylurea, the addition of SGLT2is to GLP-1RA therapy was associated with a significantly lower incidence rate of the composite cardiovascular endpoint (9.9 versus 13.0 per 1,000 person-years; adjusted pooled HR, 0.76; 95% CI, 0.59–0.98), as well as a significantly lower incidence rate of HHF (13.0 versus 20.8 per 1,000 person-years; adjusted pooled HR, 0.64; 95% CI, 0.50–0.82). The lower incidence of the composite cardiovascular endpoint appeared to be driven primarily by numerically lower incidences of myocardial infarction (adjusted pooled HR, 0.71; 95% CI, 0.51–1.00) and all-cause mortality (adjusted pooled HR, 0.68; 95% CI, 0.40–1.14) but not stroke (adjusted pooled HR, 1.05; 95% CI, 0.62–1.79) [12].

Another real-world observational study of interest was a cohort study based on the Danish National Patient Registry, which aimed to investigate the safety of the most widely used anti-hyperglycemic dual and triple therapies for T2DM [43]. There were overall 66,807 participants treated with metformin plus a combination of second- and third-line therapies. Compared with the metformin plus sulfonylurea combination, the lowest risk for all three investigated endpoints, namely MACE, severe hypoglycemia, and all cause-mortality, was seen for people treated with metformin in combination with SGLT2is and GLP-1RAs (HR, 0.53 [95% CI, 0.35–0.80] for MACE; no reported severe hypoglycemic episodes; HR, 0.18 [95% CI, 0.11–0.28] for all cause-mortality) [43].

In three nested case–control studies from England and Wales, involving 440,089 patients with T2DM and without ASCVD treated with non-insulin glucose-lowering medication, combined SGLT2i and GLP-1RA therapy used for primary prevention was associated with 30% lower odds of MACE (defined as myocardial infarction/acute coronary syndrome, stroke/transient ischemic attack, and/or cardiovascular death) compared with other combination regimens (OR, 0.70; 95% CI, 0.50–0.98) [23]. Combined SGLT2i and GLP-1RA therapy was also associated with 57% lower odds of HF when compared with other combination regimens (OR, 0.43; 95% CI, 0.28–0.64). These associations were independent of factors including ethnicity, deprivation, microvascular complications, comorbidities, HbA1c, body mass index, and the use of other medications [23].

Loyo and colleagues recently reviewed the United States Veterans Health Administration database, and identified 121,174 patients with T2DM and ischemic heart disease, cerebrovascular disease, or peripheral arterial disease who had been prescribed SGLT2is, GLP-1RAs, or their combination. Combined SGLT2i and GLP-1RA therapy resulted in a significantly lower risk of all-cause death, non-fatal myocardial infarction, and non-fatal stroke at 12 months than SGLT2i monotherapy (HR, 1.77; 95% CI, 1.43–2.20 for SGLT2i versus SGLT2i plus GLP-1RA) and GLP-1RA monotherapy (HR, 2.11; 95% CI, 1.72–2.62 for GLP-1RA versus SGLT2i plus GLP-1RA) [63].

Despite inherent limitations of observational data and potential for residual confounding, the results from these observational analyses support, in line with RCT findings, combining SGLT2is and GLP-1RAs to reduce cardiovascular events in patients with diabetes in routine clinical care [12, 23, 43, 63].

### Safety of GLP-1RA plus SGLT2i combination therapy

GLP-1RAs and SGLT2is are both generally well-tolerated when used individually, with a minimal risk of hypoglycemia, unless used in combination with insulin or insulin secretagogues [4, 9].

The most common AEs of GLP-1RAs are gastrointestinal complaints, such as nausea, vomiting, constipation, and diarrhea. However, these AEs are generally mild to moderate in intensity, and resolve spontaneously within a couple of weeks of initiating therapy [11]. A meta-analysis of 76 RCTs has recently found that compared with placebo or active controls, GLP-1RA treatment was associated with an increased risk of gallbladder or biliary diseases (RR, 1.37; 95% CI, 1.23–1.52) [64]. Similar findings were noted with dipeptidyl peptidase-4 inhibitors in another meta-analysis of 82 RCTs, in which they increased the risk of gallbladder or biliary diseases (OR, 1.22; 95% CI, 1.04–1.43) compared with controls [65]. For both therapeutic classes, this increased risk of gallbladder or biliary diseases has been thought to be driven by the inhibition of the secretion of cholecystokinin by GLP-1, leading to impaired gallbladder motility and contractility [64, 65]. Additionally, the weight loss effect of GLP-1RAs may lead to an increased risk of gallbladder disorders [64]. Overall, although the overall absolute risk increase is small (27 cases per 10,000 persons treated per year), clinical practitioners should nevertheless take into account the potential risk of gallbladder or biliary diseases associated with GLP-1RAs, especially when used at higher doses, for longer durations, and for weight loss, and in case the patient has a history of lithiasis [64].

Although an increased risk of pancreatitis is listed in product monographs for GLP-1RAs and caution should be exercised in individuals with a history of pancreatitis, causality has not been established [4, 66]. Similarly, although medullary thyroid cancer has been linked to GLP-1RAs in rodent studies, there have been very few or no reports of medullary thyroid cancer in clinical trials [4, 9, 67, 68]. A large meta-analysis of 45 RCTs recently found that compared with placebo or other interventions, GLP-1RAs did not increase the risk of thyroid cancer, hyperthyroidism, hypothyroidism, thyroiditis, thyroid mass, and goiter [69].

The SUSTAIN-6 CVOT [67], performed in 3,297 patients with T2DM and ASCVD/CKD, has shown that compared to placebo, semaglutide was associated with increased retinopathy complications (3.0% versus 1.8%; HR, 1.76; 95% CI, 1.11–2.78; p = 0.02), but mainly among those with pre-existing retinopathy at baseline [67]. However, this increased risk of retinopathy was neither replicated in other semaglutide trials nor recorded with other GLP-1RAs [70]. Of note, it is well-established that a rapid decrease in HbA1c can worsen diabetic retinopathy, particularly in patients with long-term and uncontrolled diabetes [71]. FOCUS, an ongoing, placebo-controlled, phase III study (ClinicalTrials.gov ID: https://clinicaltrials.gov/ct2/show/NCT03811561), is investigating the long-term effects of once-weekly subcutaneous semaglutide on the progression of diabetic retinopathy in individuals with T2DM.

The most common AEs of SGLT2is are genital mycotic infections [4, 11]. A meta-analysis of 77 RCTs involving 50,820 patients with T2DM found that compared with placebo, lifestyle modification, or active anti-diabetic drugs, SGLT2is were associated with a significantly higher risk of genital infections (RR, 3.30; 95% CI, 2.74–3.99), whereas no significant difference in urinary tract infections was seen between SGLT2is versus control (RR, 1.05; 95% CI, 0.98–1.12) [72]. Other AEs reported with SGLT2i use include osmotic diuresis-related events and volume depletion-related hypotension, the latter of which tends to be more common in older adults, people with an eGFR of 30–60 mL/min/1.73 m^2^, and in those taking loop diuretics [4]. Accordingly, these patients should be evaluated for orthostatic hypotension before starting therapy with a SGLT2i [11]. Euglycemic diabetic ketoacidosis is another concern that may delay initiation or result in early discontinuation of SGLT2i therapy [4, 19]. Fortunately, a meta-analysis of SGLT2i CVOTs found that diabetic ketoacidosis was rare, occurring in 0.1 to 2.2 per 1,000 person-years in those treated with a SGLT2i, albeit with a significantly greater risk compared to placebo (HR, 2.46; 95% CI, 1.43–4.24) [73]. Thus, it is recommended to investigate for this AE in SGLT2i-treated patients with symptoms of ketoacidosis, such as nausea, vomiting, abdominal pain, ketonuria, and/or ketonemia, regardless of the current glucose status [74].

Compared to placebo, canagliflozin was associated with an increased risk of lower limb amputations (6.3 versus 3.4 participants with amputations per 1,000 person-years; HR, 1.97; 95% CI, 1.41–2.75) in the CANVAS CVOT, which overall included 10,142 patients with T2DM and high cardiovascular risk [75]. However, in the other 12 CVOTs of SGLT2is, allocation to a SGLT2i was not significantly associated with lower limb amputations (RR, 1.06; 95% CI, 0.93–1.21; heterogeneity for CANVAS versus the other 12 CVOTs, p = 0.0007) [76]. These CVOT findings are supported by a real-world study performed in over 3 million patients with T2DM from the United States, in which patients treated with SGLT2is did not have a higher risk of any or lower amputation compared with incretins or other glucose-lowering agents [77].

Because the side effect profile of SGLT2is differs from that of GLP-1RAs, any additive negative interaction is not expected when these two classes of anti-diabetic medications are used in combination [11]. Moreover, evidence has shown that the AEs associated with both drug classes are largely benign in nature and relatively easy to manage [78].

In clinical studies of GLP-1RA plus SGLT2i combination therapy, the safety profile of the combination therapy was consistent with those of the individual agents, with no unexpected findings [9]. For instance, in the DURATION-8 trial [36], once-weekly exenatide in combination with once-daily dapagliflozin did not lead to major hypoglycemia episodes over 104 weeks of treatment, with few events of minor hypoglycemia (1.7%). Incidences of other toxicities were comparable across the three treatment groups in DURATION-8 (exenatide plus dapagliflozin, exenatide alone, and dapagliflozin alone, respectively), including pancreatitis (1.3%, 0.4%, and 0%), volume depletion (1.3%, 0.4%, and 2.1%), gastrointestinal events (20.8%, 23.9%, and 16.3%), and genital infections (5.2%, 2.2%, and 7.7%) [36]. In AWARD-10 [31], a 24-week, randomized, double-blind, placebo-controlled trial evaluating once-weekly dulaglutide (administered at either 1.5 or 0.75 mg) as add-on therapy to SGLT2is in a total of 424 patients with inadequately controlled T2DM, only one episode of severe hypoglycemia was reported throughout the study, in a patient treated with dulaglutide 0.75 mg, confirming the very low incidence of serious hypoglycemia with GLP-1RAs in combination with SGLT2is [31]. Across the three treatment groups (dulaglutide 1.5 mg, dulaglutide 0.75 mg, and placebo), there were no cases of diabetic ketoacidosis or amputations, and hypotensive episodes/syncope occurred in 0%, 1% and 1%, genital infections in 0%, 0% and 1%, fractures in 1%, 1% and 1%, and gastrointestinal events in 32%, 21% and 17% of patients, respectively [31].

In the previously mentioned meta-analysis by Li et al. (2022) [59] comparing GLP-1RA plus SGLT2i combination therapy to GLP-1RA and SGLT2i monotherapies among 1,895 patients with T2DM enrolled in 8 RCTs, the addition of a GLP-1RA to SGLT2i treatment showed that drug discontinuation, diarrhea, injection-site-related events, nausea, vomiting and genital infections were more likely to occur in combination therapy. Conversely, the addition of a SGLT2i to GLP-1RA treatment showed only an increased incidence of genital infections. There was no evidence demonstrating other significant safety issue differences (e.g., serious AEs) between GLP-1RA plus SGLT2i combination therapy and either GLP-1RA or SGLT2i monotherapy [59]. These data highlight the importance of closely monitoring the safety profile of GLP-1RA plus SGLT2i combination therapy in routine clinical practice.

### Profiles of patients who may benefit from GLP-1RA plus SGLT2i combination therapy

The presented cumulative evidence supports the benefits of GLP-1RA plus SGLT2i combination therapy on metabolic-cardiovascular-renal disease in patients with T2DM. Regarding their metabolic effects, the combination of a GLP-1RA and a SGLT2i significantly reduces HbA1c level, body weight, and SBP, with an additive effect on weight loss and SBP but not on HbA1c [35, 57–59]. Hence, we strongly encourage the adoption of GLP-1RA plus SGLT2i combination therapy in T2DM patients with uncontrolled metabolic risks, i.e., especially those who need to lose weight and/or control high blood pressure.

In respect to their cardiovascular effects among patients with T2DM, both GLP-1RAs and SGLT2is have been shown to reduce the risk of MACE (cardiovascular death, non-fatal myocardial infarction, or non-fatal stroke), with GLP-1RAs reducing the risk of non-fatal stroke to a greater extent [3, 6]. Thus, in perfect agreement with the recent ADA-EASD consensus report [6], we also encourage the adoption of GLP-1RA plus SGLT2i combination therapy in T2DM patients with established ASCVD (coronary artery disease, cerebrovascular disease, or peripheral arterial disease) or multiple risk factors for ASCVD (i.e., age ≥ 55 years, obesity, dyslipidemia, hypertension, current tobacco use, left ventricular hypertrophy, and/or proteinuria) whose HbA1c remains suboptimal despite initial treatment with only one of these agents.

In terms of renal effects, the evidence of SGLT2is in preventing kidney failure is more abundant than for GLP-1RAs, which showed a beneficial effect on albuminuria and not on hard kidney endpoints [3, 6, 8]. Hence, in patients with CKD and eGFR ≥ 20 mL/min/1.73 m^2^, we favor the use of SGLT2is to slow kidney disease progression [6, 27]. However, GLP-1RAs should be considered as the preferential add-on therapy in T2DM patients with CKD in case of persistent albuminuria and/or uncontrolled metabolic risks (i.e., inadequate glycemic control, hypertension, overweight/obesity).

In the setting of HF, SGLT2is reduced the risk of HHF, irrespective of ejection fraction or diabetes status [6, 28, 76, 79]. Hence, in patients with a history of HF, we encourage the administration of SGLT2is. We however think that GLP-1RAs should be avoided in people with HF with reduced ejection fraction until robust evidence of benefit is generated in this group. In the FIGHT [80] and LIVE [81] RCTs performed in patients with HF and reduced ejection fraction with and without T2DM, liraglutide treatment, compared to placebo, did not demonstrate a beneficial effect on left ventricular function, with a trend towards harm [80, 81]. Indeed, the chronotropic effects of GLP-1RAs may be deleterious in HF patients, with higher sympathetic activity contributing to morbidity and mortality [20]. In addition, frail or older patients with sarcopenia are not eligible for GLP-1RA plus SGLT2i combination therapy, as it could induce rapid and great reductions in body weight [82].

Despite the potential of GLP-1RA plus SGLT2i combination therapy to improve metabolic-cardiovascular-renal trajectories, several factors may delay this combination to become a common practice soon, and most T2DM patients with ASCVD or at high ASCVD risk remain inadequately managed. Indeed, CAPTURE, a multinational, cross‑sectional study [83] including 9823 adults with T2DM, found that less than 25% of T2DM patients are prescribed a glucose-lowering agent with proven cardiovascular benefit [83]. To the best of our knowledge, there are no published studies evaluating the cost-effectiveness of the combination of GLP-1RAs and SGLT2is. More recently, Choi and colleagues evaluated the cost-effectiveness of SGLT2is and GLP-1RAs, taken individually, as first-line therapies for American patients with T2DM, compared with metformin as first-line therapy. They found that, as first-line agents, SGLT2is and GLP-1RAs would improve T2DM outcomes, but their costs would need to fall by at least 70% to be cost-effective [84]. However, we assume that cost-effectiveness evaluations may vary from one population to another and according to the disease burden, and such findings may differ for the population for whom a combination of GLP-1RAs and SGLT2is is suitable. In some countries, reimbursement and costs associated with polypharmacy will likely be a challenge related to GLP-1RA plus SGLT2i combination therapy. In real-world practice, a SGLT2i and a GLP-1RA are more likely to be initiated sequentially rather than simultaneously, not only for economic reasons but also to improve medication adherence [1]. Hence, further investigations are warranted to advise on the combination’s cost-effectiveness in various settings.

**Fig. 2:**
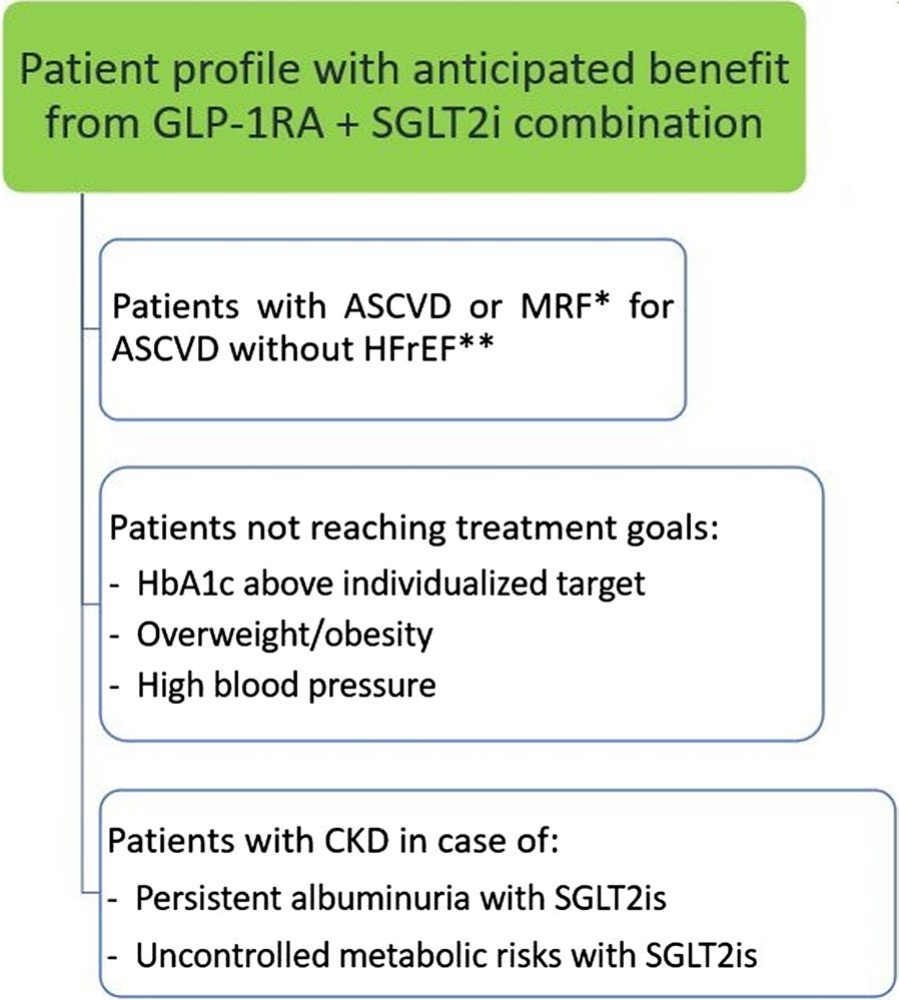
Prescribing glucagon-like peptide-1 receptor agonist (GLP-1RA) plus sodium-glucose cotransporter-2 inhibitor (SGLT2i) combination therapy in patients with type 2 diabetes mellitus. ASCVD, atherosclerotic cardiovascular disease; CKD, chronic kidney disease; HbA1c, hemoglobin A1c; HFrEF, heart failure with reduced ejection fraction; MRF, multiple risk factors. *MRF include advanced age, hypertension, dyslipidemia, smoking, obesity, proteinuria, and left ventricular hypertrophy. **GLP-1RAs should be avoided in patients with HFrEF, as they did not demonstrate a beneficial effect on left ventricular function, with a trend towards harm

Overall, when administering GLP-1RA plus SGLT2i combination therapy, it is important to adopt an individualized approach to therapy taking into account individual preferences, costs and coverage, AE profile, consideration of kidney function and glucose-lowering efficacy, desire for weight loss, and comorbidities such as frailty [6, 22, 24]. Fortunately, the combination of a SGLT2i and a GLP-1RA can be used regardless of the background anti-diabetic treatment, especially metformin, since their benefits are independent of any other drugs administered simultaneously [11]. Figure 2 suggests a decision algorithm for prescribing GLP-1RA plus SGLT2i combination therapy in patients with T2DM.

## Data Availability

Data sharing is not applicable to this article as no datasets were generated or analyzed during the current work.

## Abbreviations

ADA: American Diabetes Association
AE: Adverse event
ASCVD: Atherosclerotic cardiovascular disease
CI: Confidence interval
CKD: Chronic kidney disease
CVOT: Cardiovascular outcomes trial
EASD: European Association for the Study of Diabetes
eGFR: Estimated glomerular filtration rate
GLP-1RA: Glucagon-like peptide-1 receptor agonist
HbA1c: Hemoglobin A1c
HF: Heart failure
HHF: Hospitalization for heart failure
HR: Hazard ratio
MACE: Major adverse cardiovascular events
OR: Odds ratio
RCT: Randomized controlled trial
RR: Relative risk
SBP: Systolic blood pressure
SGLT2i: Sodium-glucose cotransporter-2 inhibitor
T2DM: Type 2 diabetes mellitus
